# SARS-CoV-2 in Danish Mink Farms: Course of the Epidemic and a Descriptive Analysis of the Outbreaks in 2020

**DOI:** 10.3390/ani11010164

**Published:** 2021-01-12

**Authors:** Anette Boklund, Anne Sofie Hammer, Michelle Lauge Quaade, Thomas Bruun Rasmussen, Louise Lohse, Bertel Strandbygaard, Charlotte Sværke Jørgensen, Ann Sofie Olesen, Freja Broe Hjerpe, Heidi Huus Petersen, Tim Kåre Jensen, Sten Mortensen, Francisco F. Calvo-Artavia, Stine Kjær Lefèvre, Søren Saxmose Nielsen, Tariq Halasa, Graham J. Belsham, Anette Bøtner

**Affiliations:** 1 Department of Veterinary and Animal Sciences, Faculty of Health and Medical Sciences, University of Copenhagen, 1870 Frederiksberg C, Denmark; hammer@sund.ku.dk (A.S.H.); mlq@sund.ku.dk (M.L.Q.); ann.sofie.olesen@sund.ku.dk (A.S.O.); saxmose@sund.ku.dk (S.S.N.); tariq.halasa@sund.ku.dk (T.H.); grbe@sund.ku.dk (G.J.B.); aneb@sund.ku.dk (A.B.); 2 Department of Virus & Microbiological Special Diagnostics, Statens Serum Institut, Artillerivej 5, 2300 Copenhagen S, Denmark; tbru@ssi.dk (T.B.R.); lolo@ssi.dk (L.L.); bstr@ssi.dk (B.S.); csv@ssi.dk (C.S.J.); frbh@ssi.dk (F.B.H.); 3 Centre for Diagnostic, Department of Health Technology, Technical University of Denmark, 2800 Kgs. Lyngby, Denmark; hhpet@dtu.dk (H.H.P.); tije@dtu.dk (T.K.J.); 4 Danish Veterinary and Food Administration, Ministry of Environment and Food, 2600 Glostrup, Denmark; stm@fvst.dk (S.M.); frafe@fvst.dk (F.F.C.-A.); stka@ssi.dk (S.K.L.); 5 Department of Infectious Disease Epidemiology & Prevention, Statens Serum Institut, Artillerivej 5, 2300 Copenhagen S, Denmark

**Keywords:** clinical signs, COVID-19, increased mortality, *Neovision vision*, seroprevalence, SARS-CoV-2 prevalence, environment

## Abstract

**Simple Summary:**

Since 2019, SARS-CoV-2 has spread and become a global pandemic. Unexpectedly, infection of farmed mink by SARS-CoV-2 was detected in the Netherlands in April 2020 and infections in three mink farms in Denmark were detected in June. Approximately 1140 mink farms were present in Denmark at the time, and a rapid increase in the number of farms with infected mink occurred from September onwards. Features of the infection on these farms were examined and potential routes of transmission between farms evaluated. It is apparent that the infection spread very easily between mink within a farm and can spread to and from people with close contact to the farmed mink. The infection spread between farms in close proximity to each other, but direct human contact is still the only identified route of virus transmission.

**Abstract:**

SARS-CoV-2 infection is the cause of COVID-19 in humans. In April 2020, SARS-CoV-2 infection in farmed mink (*Neovision vision*) occurred in the Netherlands. The first outbreaks in Denmark were detected in June 2020 in three farms. A steep increase in the number of infected farms occurred from September and onwards. Here, we describe prevalence data collected from 215 infected mink farms to characterize spread and impact of disease in infected farms. In one third of the farms, no clinical signs were observed. In farms with clinical signs, decreased feed intake, increased mortality and respiratory symptoms were most frequently observed, during a limited time period (median of 11 days). In 65% and 69% of farms, virus and sero-conversion, respectively, were detected in 100% of sampled animals at the first sampling. SARS-CoV-2 was detected, at low levels, in air samples collected close to the mink, on mink fur, on flies, on the foot of a seagull, and in gutter water, but not in feed. Some dogs and cats from infected farms tested positive for the virus. Chickens, rabbits, and horses sampled on a few farms, and wildlife sampled in the vicinity of the infected farms did not test positive for SARS-CoV-2. Thus, mink are highly susceptible to infection by SARS-CoV-2, but routes of transmission between farms, other than by direct human contact, are unclear.

## 1. Introduction

Since the end of 2019, SARS-CoV-2 has caused the COVID-19 pandemic in humans [1]. SARS-CoV-2 is closely related to that of a horseshoe bat virus (96% identical) found in China [2], although the origin has been discussed [3,4]. After entering the human population, high rates of transmission [5] and, in some cases, severe disease have been observed resulting in over 1.5 million deaths globally [6].

In experimental studies, ferrets, cats, dogs, Syrian hamsters, and non-human primates have been shown to be susceptible to the virus [7,8], and some of these species have been shown to transmit the virus under experimental conditions. In contrast, similar studies have suggested that pigs and chickens are not susceptible to infection with SARS-CoV-2 [7,8].

Denmark has been the world’s largest producer of mink pelts. The approximately 1140 mink farms, housing almost 17 million mink, produced until 2020 approximately 12–13 million pelts per year [9,10]. In addition, another 12 million pelts reared abroad were sold at the auctions at Copenhagen FUR, corresponding to a total value of approximately €700 million [9,10]. Copenhagen FUR has estimated that 6000 people were directly employed in the mink industry, with additionally thousands of people working in jobs related to the fur industry [10].

In April 2020, the first cases of SARS-CoV-2 infection in mink were detected in the Netherlands [11]. Following, these first cases, infection was also observed in mink in Denmark in June [12], in Spain in July [13], in the United States in August [14], in Italy [15] and Sweden [16] in October, in Greece [17], France [18] and Lithuania [19] in November, and in Canada in December [20]. In the Netherlands, respiratory disease and increased mortality was observed in infected farms [11].

In this study, we give an overview of the course of the SARS-CoV-2 epidemic among farmed mink in Denmark and describe the prevalence of infection, sero-prevalence, clinical signs and mortality observed in 215 infected mink farms in Denmark. In addition, laboratory results from diagnostic samples collected from these farms, together with environmental samples and samples from wildlife in the vicinity of the infected farms are described. These analyses were performed to investigate potential routes of virus transmission between mink farms. Finally, we conducted hazard analysis to investigate risk factors associated with virus detection in mink farms in the northern region of Denmark.

## 2. Materials and Methods

Farms with mink detected as infected with SARS-CoV-2 in the period from 15 June to 4 November 2020, were included in this field study. As of June 2020, SARS-CoV-2 infection in Danish fur animals has been listed as a notifiable disease. Four surveillance mechanisms were included to aid in the identification of infected farms (Table 1). Sampling on a farm was performed when (a) a person connected to a farm tested positive for SARS-CoV-2; (b) clinical signs in mink compatible with SARS-CoV-2 infection were recorded on a farm; (c) detection of SARS-CoV-2 in swab samples from dead mink on a farm taken as part of an early warning surveillance system; and (d) the farm had an association/contact to an infected farm (tracing of for example animals moved, persons working on several farms or several farms owned by the same person). Based on the regulations, all mink farms with suspicions of SARS-CoV-2 infection have to be visited by an official veterinarian, who should take samples for laboratory analysis and perform an epidemiological investigation. During this process, farmers were asked whether they were willing to participate in follow-up interviews performed by the University of Copenhagen (UCPH). Farmers agreeing to this were contacted and interviewed by telephone. Questions asked in the basic epidemiological investigation and in the follow-up interviews changed during the time of the study period, based on time constraints, as some questions were overlapping. In the descriptive analyses of the results in this paper, questions added at a later point are indicated. In the later phase of the epidemic, farmers interviewed by UCPH were asked, if they would forward their registrations of dead mink to the UCPH. The collection of mortality registrations occurred from 28 September to 4 November. Mortality in this paper indicates the numbers of dead mink over a period of time. Not all dead mink were tested for SARS-CoV-2.

The samples collected from farms with suspicion of SARS-CoV-2 infection included throat swabs and blood samples. Nasal swabs were only collected on the initial 3 infected farms. Initially, throat swabs for analysis by RT-qPCR [12] were collected at the first (suspicion) visit to the farm, and followed (if positive) by a second visit from UCPH staff, who collected further throat swabs and blood samples. As the epidemic developed, the logistics of this setup became impractical and the protocol was changed after Farm no. 35 to collect throat swabs and blood samples at the initial visit only. Due to time constraints, collection of blood samples was terminated after farm 199. During the farm visits, samples were taken from dead mink as well as from live mink. Most often, samples from 10 live mink were taken from each farm, but with some variation (1–30 samples), as well as samples from 5 to 10 dead mink. During the first (suspicion) visit, live mink with clinical signs of respiratory disease were selected for sampling. If no respiratory disease was noticed, mink from all geographical areas of the farm were selected for sampling. During the second visit from UCPH staff, mink were randomly selected for sampling. If closed houses were present, samples were taken from each of the houses, while in open sheds, animals in the periphery and in the center were randomly sampled.

Air samples were collected by UCPH staff at varying distances from mink (exhalation air directly in front of the mink, 1–2 m, 2–3 m, and >3 m) by use of AeroCollect electrostatic air samplers (FORCE Technology, Hørsholm, Denmark) for periods of approximately 5–10 min [12,21]. Furthermore, from one farm, water from the rooftop and the gutter was collected.

During the first phase of the epidemic, 89 samples were collected from the feed company that delivered feed to two of the first three infected farms. These samples, which had been stored frozen, represented batches of feed produced in the period from 1 March 2020 to 12 July 2020. From the same feed company, an additional 54 feed samples were analyzed, representing feed produced in the period from 1 July to 10 September. From eight farms (Farms 10–17), three feed samples were collected per farm during the second farm visit. In addition to feed samples, samples were obtained from two feed trucks. From one feed truck, 20 samples were collected on one specific day (24 October). Samples were collected by swabbing from wheel rims, wheel arches, metal box (on the outside of the truck, between the wheels), damper, feed delivery tube (inside, outside, edges), and driver stairs. Furthermore, feed samples were collected at the start and during the day (6 samples), 3 swabs samples were collected from the air inlet to the feed container of the truck, and 1 swab sample was collected from the filter on the air inlet. On another day (28 October), 6 samples were collected from air inlet and wheels of another feed truck, and additional feed samples were collected.

Throat swabs were collected from different species of wildlife including road kills, trapped and hunted carnivores in Northern Jutland in the period from 1 October to the end of November. Wildlife animals were only sampled, if the animals were suited for routine diagnostic necropsy. Furthermore, veterinarians in Northern Jutland collected throat swabs and blood samples from stray, feral and farm cats identified in the areas.

On farms housing other species than mink, samples from these species were collected in a few cases. Furthermore, if birds or rats were found dead or were shot at infected farms, samples from these were collected. From birds, samples were taken from feathers, throat, cloaca, and feet. Flies were collected at two farms from newly placed fly traps (sticky fly paper). Fur from mink was collected from two other farms.

Serum samples were analyzed for anti-SARS-CoV-2 antibodies using the SARS-CoV-2 Ab ELISA (Beijing Wantai Biological Pharmacy Enterprise, Beijing, China) as described by the manufacturer, while swab samples and other types of samples (e.g., flies and hair from mink) were tested for SARS-CoV-2 RNA by RT-qPCR [12].

All results are presented as simple descriptive analyses. For the sero- or virus-prevalence given, only samples from live mink are included, as the time of death for dead mink could sometimes be difficult to determine.

Hazard analysis was carried out to assess the impact of risk factors on the likelihood of contracting the disease. The potential risk factors, for which data was available for all farms, were farm size (total number of animals), feed supplier company, the veterinary practice that serviced the farm and the minimum distance to the nearest detected infected farm (MDND). For each day, the MDND was calculated as the minimum distance to farms that had been detected as having infected mink within ±14 days from a given farm. This approach was taken, because the exact infection dates for the different farms are unknown and detection was influenced by the clinical signs in the farm, the awareness of the farmer, how often persons related to the farm were tested, etc. These considerations mean that one farm could have been infected before another farm, but it could have been detected later. This variable (MDND) was included in the analyses as first and second order term following natural logarithm transformation, as this was found to provide the best model fit.

We used the date of detection of SARS-CoV-2 as the “Event”, and time from start of the suspicion date as “time to event”. The start date was set to 1 June 2020, as no mink farms were detected as infected before this date. The end date was 27 October 2020. The analysis included all affected farms in the northern region of Jutland. Farms that were depopulated during the study period were right censored. Initially, all variables were included in the model, and we then used backwards selection to remove variable individually with a *p*-value > 0.05, starting from the highest *p*-value. Thereafter, we checked the interactions between the variables in the final models and kept those where the interactions resulted in a *p*-value < 0.05. The analyses were carried out in R [22] by use of the packages “coxme”, and “survival” and “survMisc” [23,24].

## 3. Results

### 3.1. Course of the Epidemic, the Surveillance System, and the Control Measures

In total, mink from 290 Danish farms have tested positive for SARS-CoV-2. In the first phase of the epidemic in June 2020, three farms had mink that tested positive for the virus [12]; these were all located in two counties in Northern Jutland. Based on the experience from the Netherlands with SARS-CoV-2 in mink, the Danish veterinary authorities decided to cull all mink at the three farms. In the time period from 22 to 26 June, 2020, throat swabs from dead mink from 125 Danish mink farms, randomly selected from the entire country, were then collected and analyzed for SARS-CoV-2 RNA. All tested samples were negative.

**Figure 1:**
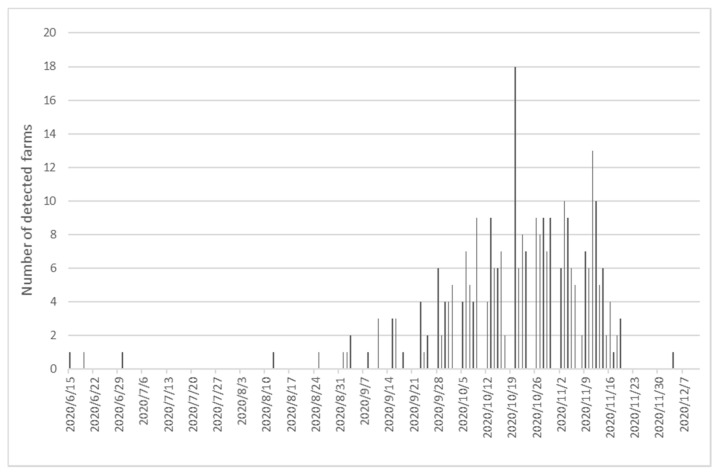
Number of Danish mink farms tested positive for SARS-CoV-2 per day in the period from 15 June to 12 November 2020.

After this phase, the epidemic went into a “silent phase”, from 4 July to 11 of August (Figure 1), where no SARS-CoV-2 infected mink were identified. During this period, a surveillance system for SARS-CoV-2 in mink was put in place, including surveillance of dead mink from all Danish mink farms every third week. Furthermore, a system was set up to survey if persons living at the same address as a registered mink farm had tested positive for SARS-CoV-2. People living on mink farms or with any other connection to mink farms were encouraged to be tested on a weekly basis. Moreover, a new regulation was put in place, which did not include culling of infected farms, but provided obligations to implement a biosecurity plan at all mink farms (positive and negative), including use of personal protective equipment while working within the animal area. On August 12, the first farm in the second phase of the epidemic was detected. This farm was also located in Northern Jutland. The suspicion was raised, based on a positive SARS-CoV-2 diagnostic result originating from the surveillance of dead mink. As a result of the new regulations, mink at this farm, and those detected subsequently, until October 8, were not culled. In the period up to September 28, the virus was detected on 30 additional farms, all located in the original two counties in Northern Jutland. From September 29, detection of infected farms occurred in neighboring counties and from October 6 more widely in Jutland. On 2 October, when 43 farms were detected positive and another 26 were under suspicion of SARS-CoV-2, it was decided to cull mink on all SARS-CoV-2 positive farms and in zones of 7.8 km around the infected farms, as laid down in Ministerial Order No. 1455 of 8 October 2020 [25]. Despite these measures, new outbreaks still occurred over the next month, and by November 4, 207 farms were tested positive and 23 farms were under suspicion; these farms were geographically spread over 20 counties in Jutland (Supplementary file, Figure S1. At this time, the Danish prime minster announced that all Danish mink, including breeding stock, should be culled and that no re-introduction of mink would be allowed before the end of 2021.

On 5 November, suspicion of re-infection with SARS-CoV-2 was raised in farm number 4 based on detection of the virus in samples collected as part of the Early Warning system based on surveillance of dead mink. This farm had been declared free from infection on 6 October, after sampling 300 mink, which corresponds to 95% certainty that a maximum of 1% of the mink were positive at that time. The farmer had noticed reduced feed intake and diarrhea in some of the mink, with no recovery following antibiotic treatment. At the time of confirmation of infection in the farm, half of the animals were already culled.

Following the decision on November 4 to cull all Danish mink, the majority of mink farms (>1100) have put down their mink (13–15 million mink in total). At the end of the epidemic and the primary culling (status as of 10 December 2020), 290 of the approximately 1140 farms (25%) had been infected.

### 3.2. Observed Clinical Signs, Mortality, and Management Factors on Infected Farms

In total, epidemiological interviews from 215 farms that tested positive for SARS-CoV-2 in the second phase of the epidemic were included in the descriptive analyses. In the beginning of October, the questionnaires were reviewed and updated, and therefore, in farms detected after 2 October, the new questionnaire was used (184 farms). Questions added after 2 October are marked in Table 2 and Table 3. A number of farms were owned by the same owner and geographically located in a way, which lead the veterinary administration to consider them as one epidemiological unit. For these farms, only one epidemiological questionnaire was completed, and therefore, there is not an absolute match between the number of outbreaks and the numbers listed in Table 1, Table 2 and Table 3.

When asked about observations of pests on the farms, a large proportion of the farmers (68%) mentioned seagulls or other birds on the farm (Table 2). Most of the Danish mink farms had feed delivered from a truck parked outside the gate, with no contact between personnel on the farm and the truck driver (Table 2). Furthermore, most farms had a changing room, and a large percentage had electric fences around the farm. All Danish establishments with >20 mink must be fenced [26]; however, there is no legal requirement to use an electric fence. Very few reported that mink had escaped within the three months prior to the interview (Table 2).

**Figure 2:**
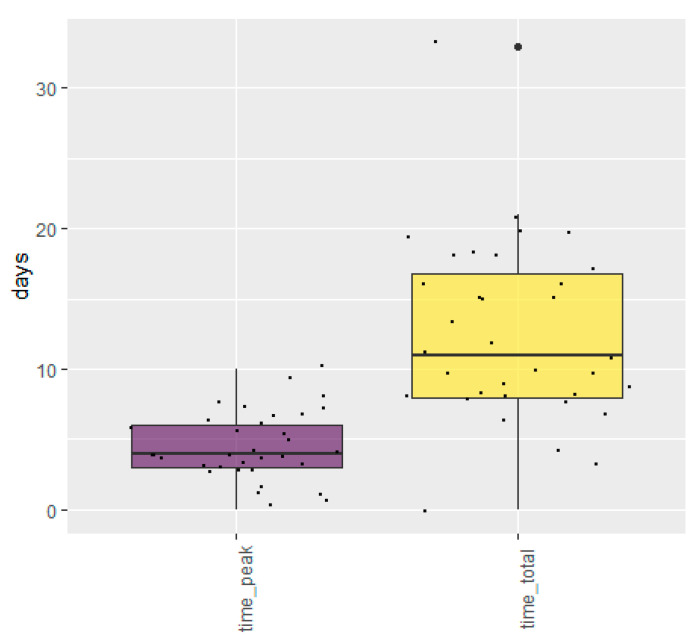
Days from when first clinical signs of SARS-CoV-2 in mink were observed to the observed peak of infection (left) or to when the last clinical signs (right) were observed by the farmer. The thick line illustrates the median, 50% of the observations are within the box, whiskers illustrates the largest value no further than 1.5 inter-quartile range from the box, and dots beyond the whiskers illustrate outliers. The small dots (jitters) within the box and whiskers indicate the observations (one for each farm).

Approximately one third of the farmers had not observed any clinical signs or increased mortality at the time of the interview (Table 3). Among the farmers observing clinical signs, reduced feed intake, increased mortality and respiratory symptoms were most common (Table 3). Of 43 farms participating in the follow-up telephone interview, 30 and 36 observed respiratory symptoms and reduced feed intake, respectively, while 24 observed nasal discharge. Furthermore, reduced feed intake was the symptom observed most commonly among the farms, most often in >20% of the mink, while nasal discharge and respiratory symptoms were generally seen in <1% of the mink (data not shown). In total, 63% of the farmers experienced increased mortality. Calculated as the number of dead mink within the last 30 days, at the time of the interview, divided by the total number of mink in the farm, the median mortality rate was 0.45% (5–95-percentile: 0.11–1.9). Based on mortality data from 30 farms, the median daily mortality rate was 0.14% (5–95th percentiles: 0.11–1.9%) on the peak day of mortality, and increased mortality was observed for a period of approximately 10 days (Table 3). From the 43 farms participating in the follow-up interviews, information on the duration of clinical signs on the farms was collected. The time from observation of the first clinical signs to the peak of clinical signs varied from 0 to 10 days, with a median of 4 days (Figure 2). Taken together, clinical signs were observed for a period of 0–33 days, with a median of 11 days (Figure 2).

### 3.3. Seroprevalence and Virus Prevalence in Infected Farms

In the first phase of the epidemic, one of the three SARS-CoV-2 identified farms (Farm 2) was detected early during the infection, with only 12.5% of throat swabs scored as virus positive (by RT-qPCR) and 3% of blood samples as seropositive on the first sampling day. This increased to 96% virus positive throat swabs four days later [12]. The other two farms were either detected with high seroprevalence (97–100%) and low virus prevalence (11%), or high seroprevalence and virus-prevalence (67% and 100%, respectively), which indicate that these farms were detected later in the course of the infection.

**Figure 3:**
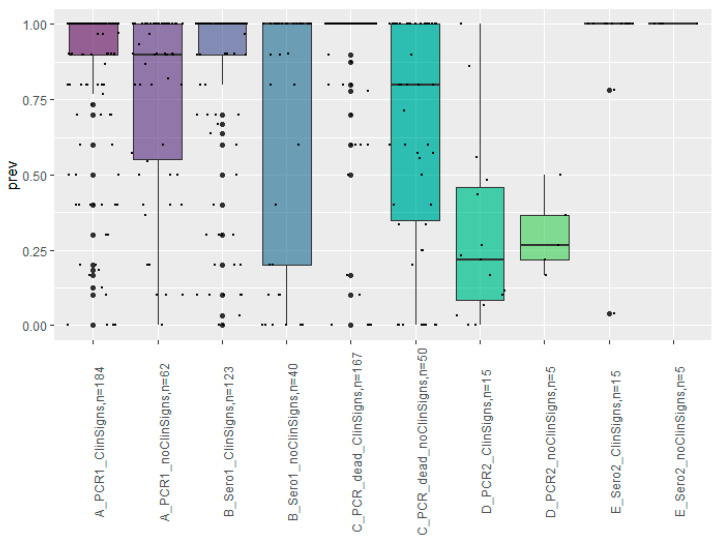
Anti-SARS-CoV-2 seroprevalence (B,E) and virus prevalence (PCR, A, C, D) in Danish mink farms in the period from 15 June to 12 November 2020, at first and second sampling dates (A, B, D, E) and virus prevalence in dead mink (C). All dead mink were from the first sampling date. Farms are divided in farms where clinical signs were observed (ClinSigns), and farms without observations of clinical signs (noClinSigns). The number of farms included in each type of sampling is indicated as n. Most often, samples from 10 live mink were taken from each farm, but with some variation (1–30 samples), as well as samples from 5 to 10 dead mink. The thick line illustrates the median, 50% of the observations are within the box, whiskers show the largest values no further than 1.5 inter-quartile range from the box, and dots beyond the whiskers indicate outliers. The small dots (jitters) within the box and whiskers illustrate the observations (one for each farm).

In later phases of the epidemic, overall 65% of the farms had a virus-prevalence of 100% at the first sampling date, while only 28 farms (12%) had a prevalence of virus positive samples below 50% on the first sampling date. In farms with clinical signs of SARS-CoV-2, 71% of the farms had a virus-prevalence of 100% at the first sampling date, while only 17 of these farms (9%) had a prevalence of virus positive samples below 50% on the first sampling date (Figure 3). Among farms without clinical signs, 45% had a virus-prevalence of 100% at the first sampling date, while 11 of these farms (18%) had a prevalence of virus positive samples below 50% on the first sampling date (Figure 3). In the second phase of the epidemic, samples were collected from 17 farms on a second sampling date. All had a seroprevalence of 100% at this time point and the prevalence of virus positive samples had significantly decreased since the first sampling date. The time between the two visits varied between 2 and 17 days, with a median of 8 days. Among the 160 farms, at which blood samples were collected at the first farm visit, 111 (69%) had a seroprevalence of 100%. In 215 farms, throat swabs were collected from 1 to 60 dead mink (median 5 dead mink). In 166 of these farms, all samples from dead mink tested positive by RT-qPCR, while in 13 farms, none of the 3–5 samples (from dead mink) tested per farm tested positive for SARS-CoV-2. The majority of the latter 13 farms had a low seroprevalence and/or virus prevalence in live mink. However, one farm had a virus prevalence of 90% in live mink and a seroprevalence of 100%, one farm had a virus prevalence of 100% in live mink but a seroprevalence of 40%, and one farm had a virus prevalence of 18% in live mink and a seroprevalence of 100%.

### 3.4. Environmental and Wildlife Samples

#### 3.4.1. Air Samples

From 19 mink farms, air samples were collected at different locations on the farm, with varying distances to mink. The number of air samples collected per farm varied from 3 to 13, with a median of 6, and the air samplers were most often running for 5–10 min (median 9.5 min). At four farms, sampling near the farm fence (>3 m from mink) occurred for 60–150 min. Out of 143 air samples in total, 22 samples from 7 different farms tested positive for SARS-CoV-2 by RT-qPCR (Table 4). Of the positive samples, 15 were collected within a distance of 10 cm from the snouts of mink. The remaining seven positive samples collected 1–2 or 2–3 m from mink were from three different farms (Table 4).

#### 3.4.2. Feed and Water Samples

In total, 174 feed samples and 19 samples taken from feed trucks were collected and analyzed by RT-qPCR. None of the samples were positive. From one farm, on two dates, swabs were taken from the roof (one sample), water dripping from the roof (one sample), the roof ridge (two samples), and gutters (nine samples), as well as water samples (three samples) from the gutters. The sample from dripping water and one of the water samples from the gutter were positive, the latter sample with three out of five replicates of this water sample testing weakly positive by RT-qPCR, the remaining two replicates tested negative.

#### 3.4.3. Wildlife

In total, samples were collected from 144 wildlife carnivores from 1 October to the end of November, including 107 red foxes (*Vulpes vulpes*), 3 badgers (*Meles meles*), 1 least weasel (*Mustela nivalis*), 3 polecats (*Mustela putorius*), 1 otter (*Lutra lutra*), 17 beech martens (*Martes foina*), and 12 raccoon dogs (*Nyctereutes procyonoides*). Furthermore, samples from 38 feral mink (*Neovison vison*) were obtained. All samples were tested negative for SARS-CoV-2 by RT-qPCR.

#### 3.4.4. Stray or Feral Cats

In total, 30 stray or feral cats (*Felis catus*) were sampled from 1 October to the end of November. All cats were tested negative for SARS-CoV-2 by RT-qPCR and by ELISA (21 of the 30 cats had blood samples taken).

#### 3.4.5. Birds

Swabs from 2 seagulls found dead on 2 different farms and from 29 birds, hereof 26 seagulls, shot on 7 other farms or in the surrounding of mink farms, were all analyzed for SARS-CoV-2 RNA by RT-qPCR. From the foot of one seagull, positive results were found, while swabs from feathers and cloaca from the same bird were negative. All other samples (114 samples in total) from seagulls and other birds (1 hooded crow (*Corvus cornix*), 1 jackdaw (*Corvus monedula*), and 1 common kestrel (*Falco tinnunculus*) tested negative.

#### 3.4.6. Other Animal Species on Detected Farms

Samples were collected from 11 dogs from 6 farms. Samples from one of eleven dogs tested positive by RT-qPCR, while three of four dogs were antibody positive in ELISA. From six farms, eight farm cats were sampled, 1 of them was positive for viral RNA.

One horse grazing between the mink cages on an infected farm was sampled and tested negative for viral RNA. Blood samples were not collected from the horse. Samples of blood, feces, and throat swabs were collected from one rat found alive in a trap on one of the farms. The samples were negative for viral RNA and for anti-SARS-CoV-2 antibodies.

From one farm with chickens, samples from seven chickens (throat and cloaca from each) were collected for RT-qPCR and two blood samples were collected. From the same farm, samples were collected from 24 rabbits (throat and rectal from each) for RT-qPCR and 18 blood samples were collected. All swabs and sera tested negative for virus and antibodies, respectively.

#### 3.4.7. Flies

Live flies were collected by hand from newly placed fly traps (sticky fly paper) from two infected farms. From one farm, 10 pools, each with three flies, were sampled on 10 October, either close to mink or approximately 25 m from mink, near but inside the fence surrounding the farm. These pools of flies were positive by RT-qPCR, but with high Ct values indicating low levels of viral RNA. Two flies collected from another farm tested negative in RT-qPCR.

#### 3.4.8. Mink Fur

Felled fur from mink was collected from two farms; in one farm fur was collected from 11 sites on the farm, while in the other farm a mixture of straw and fur was collected. All samples with fur tested positive for viral RNA; however, the samples with the highest level of virus (estimated by Ct values), were collected from the cages and in samples consisting of a mixture of fur and straw. In these samples, the levels of viral RNA were up to 100–1000 times higher compared to the maximum levels detected in samples collected 4–5 m from mink.

### 3.5. Hazard Analysis

The analysis showed that farm size and MDND were significantly associated with the chance of being diagnosed with SARS-CoV-2 (Supplementary Table S1). The interaction between these two risk factors was not statistically significant (*p*-value > 0.05). The results showed that the hazard increased with increasing farm size. The opposite was observed in relation to MDND, indicating that the closer a farm was located to a positive farm, the higher the risk of being diagnosed with SARS-CoV-2. The model explained 47% of the variation in the data.

## 4. Discussion

Among the interviewed farmers, almost 30% had not observed clinical signs in mink at the time of the interview that was carried out after the farm had been shown to be infected. If clinical signs were observed, the most common findings were reduced feed intake and increased mortality. These signs are unspecific and many farmers reported that reduced feed intake and increased mortality were often observed in late autumn before pelting the young mink. Based on the number of dead mink within the last 30 days, the median mortality rate was 0.45% (5–95-percentile: 0.11–1.9), and from the 30 farms providing detailed mortality data, the median daily mortality rate was 0.14% (5–95th percentiles: 0.11–1.9%) on the peak day of mortality. This is a clear increase, compared to data from 2002, which indicated an average weekly mortality rate of 0.008% in Danish mink farms in the period from July to December [27]. Despite the unspecific clinical signs, detection of infection based on clinical signs was the most common single marker for testing. However, in 25–28% of the infected farms detection occurred based on persons connected to the farm testing SARS-CoV-2 positive or to detection of the virus in samples collected under the Early Warning system that provided surveillance of dead mink, respectively. Already at the first sampling date, almost 70% and 72% of the farms with clinical signs had a prevalence of 100% for viral RNA and antibodies to SARS-CoV-2 (determined by ELISA), respectively, among the tested mink. This was most likely influenced by the priority of animals with respiratory signs in the sampling protocol. However, also in farms without clinical signs, 45% and 55% had a prevalence of 100% for viral RNA and antibodies to SARS-CoV-2 (determined by ELISA), respectively, among the tested mink. This could indicate either a very rapid transmission within the farm, or that the infection was detected sometime after introduction. In the Netherlands, Munnik et al. [28] estimated, based on substitution rates of SARS-CoV-2, that the infection was present for some weeks before detection in the 16 investigated farms. However, from one of the first three infected Danish farms, Hammer et al. [12] described a very fast spread in one farm with an increase in virus-prevalence from 13% to 86% in four days preceding an increase in seroprevalence from 4% to 97% in eight days. This might indicate that the virus has adapted to mink, increasing the transmission rate among mink on the farms [12]. However, even with the observed increase from 13% to 86% in four days, a slow increase in the prevalence must be expected in the first period after introduction, which implies that at least one week has passed before the first sampling date in this specific farm.

The hazard analysis showed an influence of farm size and distance to nearest infected farm. Farm size has often been shown to influence the probability of infection, as more individuals increase the probability of at least one picking up the virus, thereby initiating within farm transmission [29]. The effect of distance to nearest infected farm indicates that when virus is in the area, there is a higher risk of infection, but it does not explain how the virus was introduced to the mink farms.

In the hazard analysis, we used MDND as an explanatory variable. This covariate is time dependent as the minimum distance may vary over time, indicating that the hazard may vary over time [30]. As we do not know when the farms were actually infected, the infectious period of the mink on a farm, or the source of infection to each farm, we decided to calculate this variable as a crude value based on all farms detected within ±14 days. We acknowledge that this is not the way time-varying covariates are normally handled in hazard analyses. However, due to lack of information to allow proper handling of this variable, we used a rough and crude way of investigating the effect of distance to the nearest infected farm as a proxy; namely MDND. The 14-days value was chosen based on our experience with the speed of disease transmission through a farm. This experience was based on data from one farm that was detected early and sampled multiple times [12]. We used different cut off values in different scenarios to calculate the MDND. We observed differences in the magnitude of the MDND (results not shown), but in common for all, the MDND was statistically highly significant, as well as farm size.

Among all the different species of wildlife and animals living on the farms, SARS-CoV-2, or antibodies to it, were found in dogs, a farm cat, flies, and on the foot of one seagull. Shi et al. [8] showed that cats are highly susceptible to infection by SARS-CoV-2, while dogs have low susceptibility. However, three out of four dogs from infected Danish mink farms were antibody positive by ELISA.

Shi et al. [8] also showed that SARS-CoV-2 could spread by respiratory droplet transmission between cats in experimental settings. Furthermore, in several cases, clinical signs have been observed in cats, while, to our current knowledge, this has not been observed in dogs [31]. The dogs and cat, in which virus were detected in the present study, originated from farms where SARS-CoV-2 infection occurred in mink. In the Netherlands, SARS-CoV-2 infection was also detected in stray cats sampled from the surroundings of mink farms, either by RT-qPCR or via the presence of antibodies [11]. However, no virus or antibodies were found in feral and stray cats in Northern Jutland. By Danish law, it is required that mink farms are fenced in a way so that mink cannot pass the fence [26], and all farms met this requirement. Furthermore, half of the infected farms had electric fences, and approximately 80% had neither trees nor bushes by which the fence could be by-passed by mink and similar-sized animals. Holes were observed in 15% of the farms, and in these cases, the observation was followed by a note indicating that the holes were small and few. The lack of holes in the fences would make it difficult or impossible for wildlife to get access to the farms—depending on the animal species. These factors reduced the probability that stray cats could enter the farm areas, and thus the potential contact between stray cats and mink or cats living on the farm.

Seagulls or other birds have been described as a burden by the farmers in almost 70% of the infected farms. This is in line with the burden of birds previously described in Danish mink farms [32]. Seagulls, from a few individuals to flocks of up to 80 birds have most often been observed around Danish mink farms, but on some occasions flocks of more than 500 birds have been seen [32]. Herring gulls are most often observed on the farms. There is limited knowledge on the forage distance of these birds. However, in a study of one Danish herring gull, this bird frequently visited four mink farms within a distance of 3–4 km from its breeding ground in an observation period of two years. On 57 days, more than one mink farm was visited on the same day [32]. Most Danish mink farms consist of either solely open houses or a combination of open houses and closed buildings. Hence, birds can often easily get access to large parts of the farms. The mink feed attract birds, especially at feeding time. Seagulls are often seen foraging beneath the mink cages. Therefore, the finding of low amounts of virus on one foot of a seagull was not surprising. Generally, birds are not believed to be susceptible to SARS-CoV-2 and in experimental studies SARS-CoV-2, infection of chickens and ducks have not been demonstrated [7,8]. However, birds could act as mechanical vectors. The presence of viral RNA on fur and straw from the farms shows that the virus is present in the environment within infected mink farms. When foraging beneath the cages, birds could easily become contaminated, especially on their feet. This indicates that seagulls could potentially carry virus from one farm to another. Survival time for transmissible gastroenteritis coronavirus (TGEV) in manure has been shown to be >8 weeks at 5 °C and 24 h at 35 °C [33,34], while SARS-CoV-2 virus has been shown to survive for 28 days at 20 °C on common surfaces (e.g., glass, stainless steel, paper) [35] and for up to 9 h on human skin [36]. However, there is currently no knowledge of how long the virus is able to survive, in an infectious form, on the feet of a bird, nor on the probability of exposure from bird to mink on the “receiving” farm. Larger birds, such as seagulls, are most often observed on the roof of the farms or feeding beneath the mink cages, while smaller birds can be seen feeding on the top of the mink cages. However, based on the current knowledge, it is still difficult to determine the relative contribution of this potential transmission route.

The epidemic curve showing SARS-CoV-2 infected Danish mink farms (Figure 1) follows closely the epidemic curve of human cases seen in Denmark during the same period (https://experience.arcgis.com/experience/aa41b29149f24e20a4007a0c4e13db1d, visited 8 December 2020) with a period with few infected persons per day over the summer and a steep increase in the incidence over the autumn. Previously, transmission from humans to mink as well as from mink to humans has been shown [12,28]. Already during the silent phase in mid-summer (Figure 1), persons linked to mink farms were encouraged to be tested on a weekly basis. In addition, they were advised to wear protection masks when working on the farms and to increase biosecurity on the mink farms to avoid transmission between humans and mink. Despite these efforts, in 73% of the first 187 infected farms at least one person linked to the farms was detected as virus positive for SARS-CoV-2 (data not shown). However, further studies are needed to reveal the time from infection to detection in mink farms to be able to fully describe the nature of the transmission between humans and mink on the individual farms.

The reinfection of farm number four indicates that either virus circulation was still occurring on the farm, or a new introduction had occurred on this farm. In both August and in the beginning of October, the seroprevalence in the tested animals was 100% at the time of testing in this farm. Despite this, re-infection could indicate that not all animals became infected in August or that re-infection in November was due to reintroduction of virus. Further studies are needed to determine this.

Results obtained from questionnaires were generally the outcome of interview with the farmers and, therefore, these data rely on their interpretations and observations. Further, experience and willingness to answer these questions might influence the results. However, with the numbers of farmers interviewed, it is expected that the potential bias is limited. Samples taken from the mink indicated a very high prevalence of virus and seropositive animals among the sampled animals in many farms. These results might be influenced by the sampling strategy. When sampling was done to detect if infection was present, animals with clinical signs were most likely to be chosen. This might overestimate the true prevalence on the farms. At the first farm visit, most often 10 live mink were sampled. However, at follow-up visits 30–60 animals were sampled most often resulting in equally high prevalence.

## 5. Conclusions

After the first detection of SARS-CoV-2 in Danish mink farms in June with a small wave of 3 infected farms, 287 infected farms have been detected with a steep increase in numbers of daily detections during the late autumn 2020. Thorough epidemiological investigations in 215 of these Danish mink farms indicated that the epidemic spread quickly within the farms with a clinical picture showing respiratory signs, decreased feed intake and increased mortality for a short time period only. At the time of detection, the infection was already widespread in most of the farms. Despite sampling from numerous animals species and from the environment, no clear indicator of possible transmission routes between farms have been found. SARS-CoV-2 has been detected in air samples collected close to the snouts of mink, rarely at 1–3 m distance and in no cases beyond this distance. The virus has also been detected on fur from mink, flies, and in cats sampled on infected farms, and on the foot of one seagull. This could indicate that airborne transmission and/or birds, acting as mechanical vectors, might influence the pattern of spread seen between mink farms. However, more studies are needed to assess this. Furthermore, the hazard analysis showed that distance to the nearest detected infected farm and the farm size were strongly associated with the risk of being diagnosed with SARS-CoV-2. Still, the only clear transmission routes that have been demonstrated up until now, are transmission of virus from humans to mink and from mink to humans.

## Figures and Tables

**Table 1:** Suspicion of SARS-CoV-2 in mink based on different reporting mechanisms in 215 Danish mink farms in different periods of the epidemic.

Suspicion Based on	Period			
	16/7–6/9	7/9–4/10	5/10–13-11	Total
Clinical signs in mink	1 (14%)	26 (46%)	59 (36%)	86
Person with address on farm tested positive	4 (57%)	14 (25%)	39 (24%)	57
Surveillance of dead mink	2 (29%)	10 (18%)	52 (32%)	64
Tracing of contact to infected farm		6 (11%)	13 (8%)	19
Total	7	56	163	226 ^1^

^1^ For one farm, no cause of suspicion was given. In the total numbers, the three farms detected in June were included.

**Table 2:** Management factors registered from 215 Danish mink farms infected with SARS-CoV-2.

Observations	Yes	No	Missing	Total
Escaped mink ^1^	6	161	1	168
Seagulls or other birds ^1^	114	54	0 ^2^	168
Stray or feral cats ^1^	26	140	2 ^2^	168
Rats/mice ^1^	32	132	4 ^2^	168
Animals moved within 3 months	14	197	4	215
No. of persons on farm daily	Median: 2 (min–max: 1–8)			
Other persons on farm within 3 month period ^3^	78	131	6	215
Social contact to other mink farmers ^1^	65	96	7	168
Days since last veterinarian visit ^4^	Median: 55 (5–95-percentile: 5–118)			
Feed truck outside farm gate ^1^	150	16	2	168
Contact to feed truck driver at delivery ^1^	3	163	2	168
Visit from agricultural contractors within 3 months	26	182	7	215
Manure transport within 3 months	66	142	7	215
Delivery of manure to biogas plant	27	172	16	215
Dog on farm	74	133	8	215
Cat on farm	62	145	8	215
Entrance room ^5,6^	159	23	0	182
Entrance room clean ^5^	145	13	1	159
Entrance room used ^5^	151	7	1	159
Electric fence ^4^	90	90	2	182
Access over fence ^5^	31	150	1	182
Holes in gate ^5^	28	153	1	182

^1^ Question was added to the questionnaire in beginning of October. ^2^ This question was phrased “Have pests (rats, seagulls, foxes) been observed on the farm?” In the descriptive analyses, we divided the answers into seagulls and other birds, stray and feral cats and rats/mice, and groups not mentioned by the farmer were considered as a “No”-answer. ^3^ This is a combination of a question on whether there had been contact to other mink farms and whether there had been any visitors. ^4^ Answer was “start, middle or end of…” were registered as 1st, 15th, or 30th of the given month. ^5^ Question answered by visiting veterinarian. This part of the questionnaire was received from 182 farms. ^6^ An area where the personnel and visitors could change clothes and boots etc. For farms without entrance rooms, answers on whether the room was clean and in use were not included in the analyses.

**Table 3:** Clinical signs and mortality observed by farmers in 215 ^2^ Danish mink farms tested positive for SARS-CoV-2 in different periods of the epidemic.

Observations	Period			
	16/7–6/9	7/9–4/10	5/10–13/11	Total
Number of mink farms detected ^1^ in the period	7	56	212	275
Number of farms with clinical signs	Yes/Total (%)	Yes/Total (%)	Yes/Total (%)	Yes/Total (%)
Number of mink on farm, Median (5–95-percentile)	15,000 (5269–24,745)	11,550 (2825–36,700)	12,065 (3323–35,317)	12,100 (3200–35,788)
Clinical signs, in adults	4/7 (57%)	30/55 (55%)	75/145 (52%)	109/207 (53%)
Clinical signs, in young (kits)	3/7 (43%)	38/55 (69%)	83/145 (57%)	124/207 (60%)
No clinical signs	3/7 (43%)	16/55 (29%)	43/145 (30%)	62/207 (30%)
Mortality % (deaths within 30 days/number of mink), Median (5–95-percentile)	0.39% (0.17–0.49)	0.38% (0.036–1.04)	0.5% (0.13–2.3)	0.45% (0.11–1.9)
Increased observed mortality	4/7 (57%)	27/55 (49%)	103/150 (69%)	134/212 (63%)
Nasal discharge	1/7 (14%)	16/52 (31%)	35/141 (25%)	52/200 (25%)
Sneezing	0/7 (0%)	13/52 (25%)	33/141 (23%)	46/200 (23%)
Respiratory symptoms	2/7 (29%)	14/52 (27%)	46/141 (33%)	62/200 (31%)
Depression	1/7 (14%)	5/52 (10%)	8/141 (6%)	14/200 (7%)
Reduced feed intake	1/7 (14%)	32/52 (62%)	74/141 (52%)	107/200 (54%)
Diarrhea	0/7 (0%)	5/52 (10%)	5/141 (4%)	10/200 (5%)

^1^ Date of suspicion is used as detection date, to avoid influence of delayed detection caused by potential limitations in resources for surveillance visits. ^2^ Not all farmers answered all questions, and therefore the total does not sum to 215.

**Table 4:** Results of PCR analyses of SARS-CoV-2 in air samples collected in 16 Danish mink farms at varying locations.

Distance from Air Sampler to Mink	Positive Samples (Farms)	Negative Samples (Farms)	Total Samples (Farms)
<10 cm	15 (7)	44 (17)	59 (19)
1–2 m	4 (3)	44 (17)	48 (18)
2–3 m	3 (2)	7 (6)	10 (8)
>3 m	0 (0)	26 (17)	26 (17)
Total	22 (7)	121 (19)	143 (19)

## Data Availability

The data presented in this study are available on request from the corresponding author.

## Supplementary Materials

The following are available online at https://www.mdpi.com/2076-2615/11/1/164/s1, Table S1: Results of the hazard analysis for SARS-CoV-2 infection being detected in mink farms in Northern Jutland during June to October 2020, Figure S1: Animation of geographical distribution of Danish farms with detection of SARS-CoV-2 in mink based on official detection date. (GIF-file).
