# Supplementary material for: SARS-CoV-2 in Danish Mink Farms: Course of the Epidemic and a Descriptive Analysis of the Outbreaks in 2020
Source: Animals (Basel). 2021 Jan 12;11(1):164. doi: 10.3390/ani11010164 (PMC7828158; doi:10.3390/ani11010164)
Supplement: Supplementary file 1 [file animals-11-00164-s001.zip › animals-1062435_Supplementary Table S1.docx]

Supplementary:

**Table S1: Results of the hazard analysis for SARS-CoV-2 infection being detected in mink farms in Northern Jutland during June to October 2020.**

| Risik factor |  | Coefficient | Hazard ratio | P-value |
| --- | --- | --- | --- | --- |
| Farm size |  | 0.000029 | 1.000029 | <0.0001 |
| Log(MDND) |  | -21.53 | 4.45*10^-10^ | <0.0001 |
| (Log(MDND))^2^ |  | -9.176 | 1.45*10^-4^ | <0.0001 |
