# Supplementary figures and images for: SARS-CoV-2 in Danish Mink Farms: Course of the Epidemic and a Descriptive Analysis of the Outbreaks in 2020
Source: Animals (Basel). 2021 Jan 12;11(1):164. doi: 10.3390/ani11010164 (PMC7828158; doi:10.3390/ani11010164)

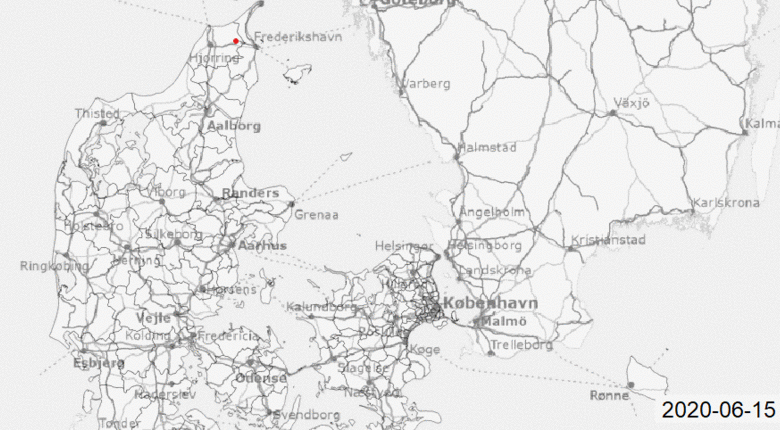

Supplement: Supplementary file 1 [file animals-11-00164-s001.zip › Supplementary Figure S1.gif]
